# Parental Perceptions of Priorities and Features for a Mobile App to Promote Healthy Lifestyle Behaviors in Preschool Children: Mixed Methods Evaluation

**DOI:** 10.2196/65451

**Published:** 2025-02-19

**Authors:** Jessica R Thompson, Summer J Weber, Shelagh A Mulvaney, Susanna Goggans, Madeline Brown, Anthony Faiola, Lynn Maamari, Pamela C Hull

**Affiliations:** 1 Department of Health Policy and Administration The Pennsylvania State University University Park, PA United States; 2 Community Impact Office University of Kentucky Markey Cancer Center University of Kentucky Lexington, KY United States; 3 Evidence Analysis and Regulatory Affairs Food and Nutrition Service United States Department of Agriculture Alexandria, VA United States; 4 School of Nursing Vanderbilt University Nashville, TN United States; 5 Department of Population Health College of Nursing University of Cincinnati Cincinnati, OH United States; 6 Department of Behavioral Science University of Kentucky Lexington, KY United States

**Keywords:** mHealth, childhood obesity, mixed methods, pediatric, healthy lifestyle behaviors, preschool children, mobile application, diet, physical activity, exercise, media use, sleep, development, semi-structured interviews, healthy eating, parents, caregivers

## Abstract

**Background:**

Parents of preschool-aged children are a key focus for interventions to shape healthy lifestyle behaviors and support risk reduction for obesity from an early age. In light of limited existing evidence on the use of mobile technology to promote healthy lifestyle behaviors among young children, we sought to gather parental priorities regarding a mobile app focused on guided goal setting across the domains of diet, physical activity, media use, and sleep.

**Objective:**

The purpose of this study was to explore the priorities and needs of parents of 2- to 5-year-old children to guide developing the content and features of a mobile app aimed at promoting healthy lifestyle behaviors using a novel convergent mixed methods approach.

**Methods:**

From November to December 2021, we invited parents or guardians in Kentucky to complete a series of web-based concept mapping activities and semistructured interviews (total N=30). Using 2 lists of items focused on (1) parental priorities (content areas) and (2) application features, we asked participants to conduct concept mapping procedures for each list: a web-based sorting activity, where participants grouped items together into thematic piles that made sense to them, and a rating activity, where participants rated each item on a 5-point Likert-type scale. The qualitative interviews were transcribed verbatim, coded, and then analyzed by constant comparative analysis to identify themes. We used the quantitative findings from the concept mapping process to triangulate the resulting themes from the qualitative interviews and generate possible app content areas and features.

**Results:**

The concept mapping results resulted in two 3-cluster concept maps. For parental priorities, participants identified the clusters Creating Healthy Eating Habits, Forming Boundaries, and Building Good Relationships; for app features, participant clusters included Eating Healthy, Using the App, and Setting Goals. The interview themes also represented those 2 domains. Overall, the participants indicated that the top priorities were general health and wellbeing, routine and setting boundaries, and food and healthy eating when it comes to building healthy behaviors among their preschool-aged children. Parents indicated that quick, easy, and child-friendly recipes, goal tracking, and the use of tips and notifications were the features they valued most.

**Conclusions:**

This study contributes to the understanding of what parents or caregivers of young children want from mobile apps, in both content and features, to support building healthy behaviors and routines. The findings can inform future research on the development and evaluation of existing or new mobile apps. Specific app features identified to meet family needs should be designed closely with a diverse set of families and tested using rigorous designs to identify the mechanisms of action that mobile apps may use for efficacious healthy parenting outcomes.

## Introduction

Healthy lifestyle behaviors remain an essential focus for health care efforts in the United States. As of 2017-2020, the US obesity prevalence rate was 41.9%, with obesity being the leading cause of preventable death across major outcomes, such as heart disease, stroke, type II diabetes, and several forms of cancer [1]. Likewise, childhood obesity rates continue to be a concern in the United States, including a prevalence rate of 12.7% among 2- to 5-year-old children in 2017-2020, resulting in conditions such as high blood pressure, high cholesterol, asthma, sleep apnea, and joint problems at an early age [2]. Additionally, childhood obesity disproportionately affects children of lower socioeconomic status and minority groups [3-5], exacerbating and contributing to this vast array of secondary consequences [6,7]. The state of Kentucky currently ranks second in the nation for both adult and childhood obesity rates, with significantly high rates of obesity among rural Appalachian and urban minority residents [1,2,8]. Parents of preschool-aged children remain a key focus for interventions supporting communities with disproportionately high obesity rates in an attempt to curtail these rates from an early age [9].

Recent research on healthy lifestyle behaviors among preschool-aged children has identified relevant determinants and behaviors. For example, Townsend et al [10,11] describe 12 dietary and behavioral determinants for childhood obesity risk reduction: fat, dietary fiber, fruit or vegetables, calcium or dairy, sweetened beverages, restaurant-prepared food, breakfast, energy density of eaten foods, physical activity, TV-viewing, sleep duration, and parenting. A recent systematic review of family-based childhood obesity prevention interventions indicates that interventions tend to focus on the domains of diet, physical activity, media use, and sleep; however, less than half of the included studies targeted a behavioral domain beyond diet and physical activity, and only 16% targeted all 4 behavioral domains [12]. Likewise, few studies exist that focus on goal-setting interventions among children and adolescents [13], even though guided goal setting represents a feasible strategy for parents to improve health behaviors for obesity risk reduction among children [14]. Additionally, a recent meta-analysis indicated that obesity prevention interventions in youth with low socioeconomic status are more successful when several behavior change strategies are integrated, rather than relying solely on one strategy [15].

Thousands of mobile apps exist that focus on nutrition and physical exercise but are primarily geared toward adults [16]. In our recent review, we found only 3 preexisting reviews of multiple mobile health (mHealth) apps designed for children and parents between 2015 and 2022 [17]. Of the 3 reviews, only one provided a list of specific app names, focusing on nutrition as a behavioral intervention for child obesity [18]. The authors of this review concluded that most apps, although free or relatively inexpensive (making them highly attractive to parents), contained content of low quality, were poorly designed, or were not grounded in credible dietary guidelines due to a lack of involvement of nutrition or scientific professionals in the design process [18]. In this review, the most common nutrition features in such apps included promotion of energy balance and guidance on appropriate portion size, and the most common behavioral change feature involved goal setting. Our review identified only 9 apps that use goal setting to change the health behaviors of children.

In light of limited existing evidence, we sought to design a mobile app geared toward parents of preschool-aged (2- to 5-year-old) children, integrating guided goal setting across the domains of diet, physical activity, media use, and sleep. During the formative stage of app development, we sought to integrate feedback from parents with children in our target age range in order to assess major areas of interest and ways the app could best meet their needs. Using a novel mixed methods approach, we combined concept mapping activities and qualitative interviews to capture the perceptions of parents on app content and features. Concept mapping is a useful formative design research tool that uses a structured process to create a visual representation or cognitive map of ideas and concepts [19]. This method has been widely used in a range of formative research topics including mHealth app evaluation [20] and in the development of apps focused on reducing school absences [21]. To our knowledge, this is the first application of concept mapping combined with interviews in the formative design and development of a health-focused app for parents of preschool-aged children. Specifically, the purpose of this study was to use this novel convergent mixed methods approach to explore the needs and desires of parents of 2- to 5-year-old children for the content and features of a health-based mobile app designed to promote healthy behaviors to reduce future obesity risk.

## Methods

### Recruitment

Study inclusion criteria include the following: primary caregiver of a 2- to 5-year-old child, 18 years or older, and use a smartphone or mobile phone and smartphone apps. Participants were recruited via printed flyers placed at various community-based organizations and distributed via community outreach activities as well as email listservs and social media channels managed by the university. Recruitment advertisements invited parents to participate in a study to help design a smartphone app for families. Those interested in the study could connect with the study team via an internet link, email, or telephone. The study team employed eligibility prescreening via a REDCap (Research Electronic Data Capture; Vanderbilt University) survey [22]. Participants had the option to complete the eligibility survey online or over the phone.

### Ethical Considerations

A waiver of documentation of consent was granted by the University of Kentucky Medical Institutional Review Board (61563). The survey included a web-based process with consent indicated by selecting the “submit” button. If administered by phone, the staff read the consent information and asked participants to consent verbally. Eligible participants were contacted by study staff to schedule an interview and sent a link and log-in information for concept mapping activities.

### Study Procedures

From November to December 2021, we conducted 60-minute qualitative interviews (N=30). Interviews were facilitated by staff with a background in public health nutrition and qualitative methodology and conducted via Zoom (Zoom Video Communications) [23]. Interviews were recorded with participant permission. The interview protocol was developed based on a multilevel model of behavioral factors related to pediatric overweight (eg, dietary intake, physical activity, sedentary behavior, sleep, family meals, parenting styles, and feeding practices) [10,11] and research on the importance of goal setting in behavior change [24,25]. In addition, questions were informed by our formative research that demonstrated interest in health-focused apps designed among parents with preschool aged children [26,27] and a review of the content and features of existing apps for parents related to goal setting and tracking [17]. Based on this literature, we developed a semistructured discussion guide (Table S1 in Multimedia Appendix 1) in which we intentionally grouped questions in three logically progressing areas: (1) overall parenting routines and challenges, (2) health and wellness for their preschool-aged child with probes in specific domains from the literature (eg, mealtime, sleep, and active play), and (3) current mobile app use and desired features.

Using a convergent mixed methods design [28], interview participants were simultaneously asked to join in web-based concept mapping activities. Concept mapping is a participatory mixed method that incorporates quantitative elements with qualitative data collection to build consensus around a topic of interest [19,29]. To begin, the study team developed 2 sets of items based on formative research [26,27], which responded to two focal questions: (1) What are health and wellness priorities of your 2- to 5-year-old child? (23 items) and (2) What are the features you would like to have on an app on your phone that could help you set goals and work on goals for taking care of the health and wellness of your 2- to 5-year-old child (31 items)? The first question sought to gather perspectives on app content while the second served to capture desires for app features. We asked participants to complete 2 procedures: a virtual sorting activity, where participants grouped items into thematic piles that made sense to them, and a rating activity, where participants rated each item on a 5-point Likert-type scale. For the 23 health and wellness priority items, this rating question was as follows: How important is this item to you as a priority for your child's health (from not at all important [1] to extremely important [5])? For the 31 app feature items, we asked the following question: How often would you use this feature in an app on your phone (from never [1] to all the time [5])? In addition, we asked demographic questions, including race, ethnicity, marital status, number of children, employment status, education, income, and receipt of assistance programs. All concept mapping data were collected using Groupwisdom (Concept Systems, Inc), a web-based concept mapping software [30].

### Analysis

The qualitative interviews were transcribed verbatim and analyzed through a combination of a deductive approach and grounded theory to identify themes [31,32] using the ATLAS.ti qualitative data analysis software (version 8.0) [33]. The initial identification of overarching thematic codes used a deductive strategy based on the interview questions, which was informed by previous research on behavioral determinants related to childhood obesity risk reduction, mealtime and child feeding practices, and guided goal setting among parents of preschool-aged children [10,11]. This initial deductive analysis also focused on overarching thematic codes related to potential app features and tools for families of preschool-aged children [17]. Additionally, we used a line-by-line analysis approach to generate a list of emergent, more granular subcodes within the larger coding schematic. In total, 3 coders with backgrounds in public health were trained to allocate codes to quotations. Interrater reliability was determined according to the procedures of Gough and Conner [34], which resulted in a high level of correspondence (93% agreement). The criteria of Lincoln and Guba [35] for trustworthiness of qualitative research were applied to ensure credibility of the findings.

We analyzed the sorting data from each set of concept mapping items in the Groupwisdom software. Specifically, we used similarity matrices and multi-dimensional scaling to generate 2-point maps, which reflect group consensus on the similarity of items. Next, we performed hierarchical cluster analysis for each set of sorting data to group the sets of items into common thematic clusters, resulting in 2 cluster maps. We combined the point and cluster maps for each focus area (parental priorities and app features) to depict items within their thematic areas. As a part of the recommended concept mapping methodology, we also analyzed average item ratings for the Likert-type rating scales to determine which items participants believed were the most important priorities for the health and wellness of their child and which app features were most likely to be used [19].

Finally, we used the interview themes to triangulate the concept mapping findings. Specifically, we synthesized the parental priorities and desired app features results to derive possible content and features of a novel mobile app to improve health behaviors among preschool-aged children.

## Results

### Participant Characteristics

Table 1 shows the characteristics of those who participated in at least one activity (N=30): qualitative interviews only (n=2), concept mapping only (n=4), or both (n=24). Overall, the majority of participants identified as non-Hispanic White (93.3%), married (70.0%), having 1 or 2 children (80.0%; range 1 to ≥4), and being employed either part- or full-time (63.4%). A significant portion of the sample (36.6%) had a monthly income of US $2999 or less and 40.0% had not obtained a Bachelor’s degree. For families using public assistance, most participated in a combination of WIC (Special Supplemental Nutrition Program for Women, Infants, and Children), SNAP (Supplemental Nutrition Assistance Program), and Medicare or Medicaid.

**Table 1:** Characteristics for interview or concept mapping participants (N=30).

Demographic variables		Frequency, n (%)
**Race or ethnicity**		
	Non-Hispanic White or Caucasian	28 (93.3)
	Hispanic, Latino, or Spanish origin	2 (6.7)
**Marital status**		
	Married	21 (70.0)
	Single—divorced	2 (6.7)
	Single—never married	6 (20.0)
	Did not respond	1 (3.3)
**Children**		
	1	14 (46.7)
	2	10 (33.3)
	3	3 (10.0)
	4 or more	3 (10.0)
**Employment**		
	Full-time	17 (56.7)
	Part-time	2 (6.7)
	Not employed	11 (36.7)
**Education**		
	High school or GED (General Educational Development high school equivalency diploma)	3 (10.0)
	Some college or technical degree	9 (30.0)
	Bachelor's degree	5 (16.7)
	Graduate degree	9 (30.0)
	Did not respond	4 (13.3)
**Monthly income**		
	<US $1000	4 (13.3)
	US $1000-US $1999	3 (10.0)
	US $2000-US $2999	4 (13.3)
	US $3000-US $3999	1 (3.3)
	US $4000-US $4999	5 (16.7)
	US $5000+	7 (23.3)
	Did not respond or don't know	6 (20.0)
**Types of assistance**		
	WIC (Special Supplemental Nutrition Program for Women, Infants, and Children)	8 (26.7)
	Head start	2 (6.7)
	SNAP (Supplemental Nutrition Assistance Program)	7 (23.3)
	TANF (Temporary Assistance for Needy Families)	0 (0.0)
	Medicare or Medicaid	6 (20.0)

### Concept Mapping and Interview Results

The concept mapping results reflected both app content and features: parental priorities and app features. The in-depth interviews resulted in thematic areas containing 257 individual total codes which also fell into these areas. The parental priority themes included the following: desired areas for improvement, routine, mealtime or child feeding behaviors, active play or physical activity, and sleep. The app features’ themes included recipes, goal tracking, grocery shopping or meal planning, and tips and notifications. Representative quotes from the identified themes are shared in Table 2.

**Table 2:** Representative quotes from the in-depth interviews by thematic areas.

Themes		Quotes
Desired areas of improvement		I care about, of course, my children and their well-being. That they stay happy and healthy; mentally, physically, emotionally. (Mother of 2 boys ages 3 and 6 years old) I'm always up for changes, improvements, anything that I can do that'll make anything easier or more efficient because a lot of times I’ll do things the hard way instead of an easier way. I don’t know why. My dad always said that I do everything the hard way. (Mother of a 4-year-old boy and a 1-year-old girl)
Routine		We do try to follow [a routine] just because it tends to let our girls have a better, you know, day, especially when they're on their routine- and if they're not in routine, they tend to act out more. So, we do try to... but we don't always follow it, and sometimes it bites us in the [expletive] so on those days it's a little more difficult. (Mother of 2 girls 7 and 2 years old)
Mealtime and child feeding behaviors		She's very picky. And I have a lot of issues with my weight and I'm afraid that she's going to too. So, I try to like- “Oh well here, I'm eating broccoli. Do you want to eat this? Because dinosaurs eat broccoli” because she likes dinosaurs so she's like, “No it's OK- I'm a dinosaur that doesn't like broccoli.” (Mother of 2 girls ages 6 and 4 years old)
Planning grocery shopping		I do better if I have a list. Sometimes we'll make a list on my phone before I go into the store, so I have something to make sure I get everything. And then a lot of times we'll use Click List, which makes it a lot easier because then everything that we need is all right there, like we have to order it that way, so that makes it a lot easier[…] Sometimes I'm still a pencil paper person too. I kind of do all of it. (Mother of a 4-year-old boy and a 1-year-old girl)
Active play or physical activity		Then I know at school, right now they only get to go outside if it's warm enough so he hasn't been outside much, and I can tell on the days when he's been able to play outside at school versus when he hasn't. (Mother of a 3-year-old boy)
Sleep		They report his behavior at school because sometimes he does not get good reports and his behavior at school- and I've told them it is directly related to sleep. […] That's one of the biggest factors like nights where I know that he's not slept as much, or if they've said he's took a nap or not. It's most directly related to his bad behavior, a lot of times at school, it'll be one the number one thing. So that is important. (Female caregiver to a 4-year-old boy)
**App features**		
	Recipes	The recipes that get more attention for me are ones I might actually use instead of just browsing would be the ones that's catered for the kids. Or to make it better for them or like a kid twist on something you know, that would be what I would want. (Caregiver to a 4-year-old boy)
	Goal tracking	I know at home sometimes we'll set goals for him. If there's an area that we see, an area of concern, then we'll do like a sticker chart like that I would use in school with a student to meet a certain goal, and we'll talk about that you have to do these three out of the five times. And so, I think if there was an app that would probably be easier than creating sticker charts all the time. (Mother of a 5-year-old boy)
	Grocery shopping and meal planning	I would be interested in it [a shopping list feature] if it was kid friendly and simple. Like if it would be probably like the simplest and quickest kind of interface. Some have gotten to cluttered and have to many things going on, to many things to edit, so, simple is best. (Caregiver to a 4-year-old boy)
	Tips and notifications	I would just say simple. As a parent with everything else on my plate, simplicity is the easiest way[…] if I’m looking for something in particular, I want to be able to find it as quickly as I can. (Mother of 2 boys ages 2 and 4 years old)

#### Parental Priorities

**Figure 1:**
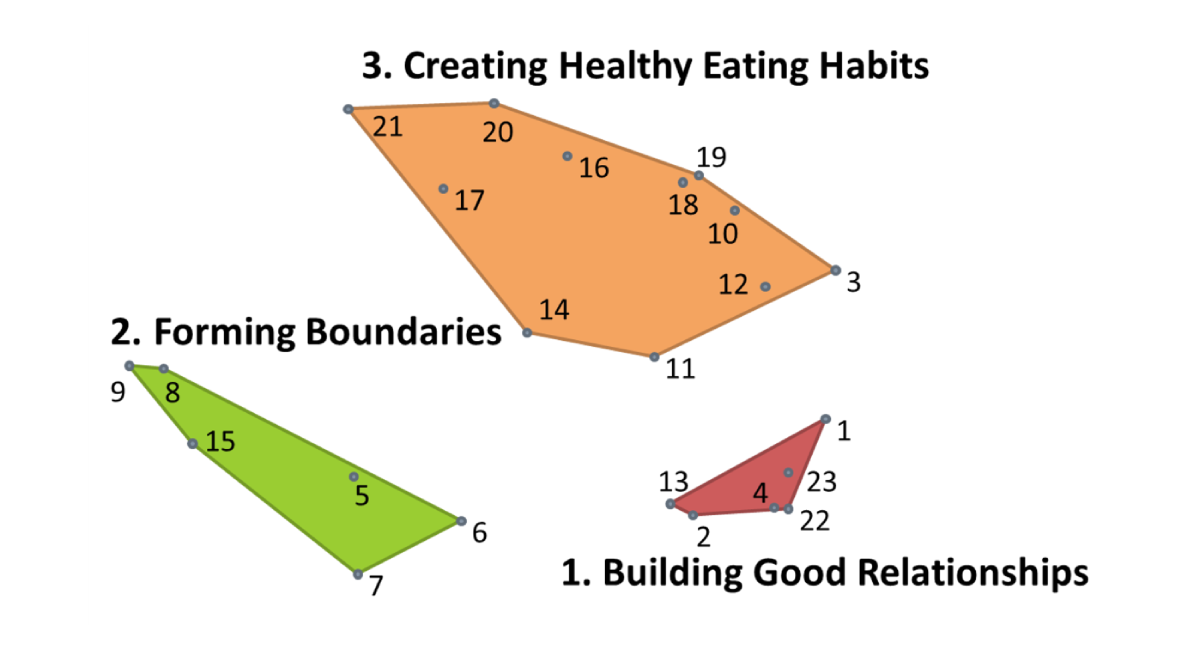
Parental priorities combined point and cluster concept map.

Figure 1 displays the combined point and cluster map for the parental priorities area, which shows each of the 23 items in relative positions based on similarity (ie, items close together were often sorted together by participants) and grouped into thematic clusters, providing understanding of perceived commonality. The resulting clusters were as follows: (1) Building Good Relationships, (2) Forming Boundaries, and (3) Creating Healthy Eating Habits. These cluster names were developed from pile names participants created in the sorting process and reflect the major desired areas for app content. Table 3 includes all of the concept mapping items grouped by cluster and sorted by the average rating for each item; the highest rated items for each cluster are shown in italics.

**Table 3:** Concept mapping items grouped by cluster with average item ratings (highest rated for each cluster in italics).

Cluster and item			Statement	Average rating (Importance: 1=not at all to 5=extremely)
**Parental properties**				
	**1. Building good relationships**			
		*1*	*Taking care of my child's health*	*4.96*
		*22*	*Being a good parent*	*4.92*
		2	Talking and listening to my child	4.88
		23	Helping my child feel good about their body as they grow up	4.88
		4	Spending quality time with my child	4.79
		13	Playing with my child	4.67
	**2. Forming boundaries**			
		*6*	*Setting and sticking to boundaries that are clear*	*4.50*
		*5*	*Establishing and sticking to a daily routine*	*4.17*
		7	How to reward my child for good behavior	4.08
		8	Limiting my child's time using a phone or tablet	4.00
		15	Getting my child to bed on-time	4.00
		9	Limiting my child's time watching TV	3.67
	**3. Creating healthy eating habits**			
		*3*	*Having a variety of healthy food choices at home*	*4.63*
		*12*	*Being a good role model for my child by eating healthy*	*4.42*
		19	Getting my child to eat vegetables	4.38
		18	Getting my child to eat healthy food	4.29
		11	Encouraging exercise or physical activity for my child	4.25
		10	Making sure my child doesn't drink too much sugar	4.13
		14	Being a good role model for my child by exercising	4.04
		16	Preparing more meals at home	3.92
		17	Cooking with my child	3.67
		21	Ways to address my child's picky eating	3.58
		20	Quick and easy recipes for my family	3.50
**App features**				
	**1. Eating healthy**			
		*1*	*Healthy recipes*	*4.42*
		*4*	*Easy recipes*	*4.29*
		*5*	*Quick recipes*	*4.29*
		3	Recipes for kids	4.17
		11	Add recipes to a meal plan	4.08
		8	Recipe tips	4.04
		6	Search and filter recipes	3.92
		7	View recipe nutrition facts	3.88
		10	Make a meal plan	3.83
		12	Tips about child health and nutrition	3.79
		9	Make a grocery list	3.71
		2	Recipes that can fit my family’s dietary needs (eg, vegetarian, gluten free, no dairy, allergies, etc.)	3.50
	**2. Using the app**			
		*13*	*App alerts to view tips about child health and nutrition*	*3.50*
		*31*	*Links to videos about child health and nutrition*	*3.50*
		*23*	*Configure app preferences*	*3.25*
		22	Setup my family profile—selecting a photo or avatar	2.88
		28	Using the app with other family members	2.54
		26	Sharing my goal progress with friends and family	2.08
		25	Sharing my goal progress with other app users	2.04
		27	Sharing my goal progress on social media	1.83
	**3. Setting goals**			
		*20*	*Set and track goals about making food more child friendly*	*3.88*
		*30*	*Rewards or points for reaching my goals*	*3.79*
		14	Quizzes to assess my child’s health and well being	3.63
		29	Getting feedback on how to improve on my goals	3.50
		15	Set and track goals about my child’s health and well being	3.42
		24	Set and track goals about what my child eats and drinks	3.42
		16	Set and track goals about my child’s physical activity	3.33
		18	Set and track goals about my child’s mealtime routine	3.33
		21	Reminder alerts to track goals for my child daily	3.29
		19	Set and track goals about eating together as a family	3.21
		17	Set and track goals about my child’s bedtime	3.13

### Building Good Relationships

Items in the Building Good Relationships cluster with the overall highest importance ratings included: “1. Taking care of my child’s health” (4.96 out of 5) and “22. Being a good parent” (4.92 out of 5), which highlights the importance of overall child well-being. To begin the interviews, we asked about what is most important as a parent or caregiver of your 2- to 5-year-old child or children. Several parents mentioned child happiness or child emotional or mental health as a main priority. Parents also mentioned spending time with their child, active listening, and communication with their child as important aspects of their parenting style. Many parents stated their child’s health in general was most important, while other parents mentioned specific areas of child health such as healthy eating and physical activity. Other areas of priority included building and sticking to a routine, school readiness, and participation in religion.

### Forming Boundaries

The Forming Boundaries cluster spoke to the participant-identified need for routine and time management. Items “6. Setting and sticking to boundaries that are clear” (4.50 out of 5) and “5. Establishing a daily routine” (4.17 out of 5) had the highest importance ratings in the cluster. Caregivers described a routine or schedule to establish consistency and reliability for their children. The morning routine, although often the most stressful or hectic, was integral to several families, especially those with a strict morning schedule. Evening routines were often described as a way to establish togetherness and connection with their children, whether through mealtime, playtime, or screen time. Bedtime routines varied for families but were the most commonly described area of routine. Caregivers described that their schedules were highly dependent on a variety of factors such as non-autonomous work schedules, unemployment, appointments, busy lifestyles, child health, and their child’s mood or behavior.

Many of those who said that their routine was important or somewhat important emphasized that “life is easier with routine, more difficult without,” and that their children were motivated by or thrived on routine. However, not all caregivers identified as planners; ie, several caregivers explicitly mentioned they do not adhere to a routine or *play it mostly by ear.* Whether parents viewed routine as important or not, they were able to describe scenarios where lack of structure was linked to child behavior issues.

Appropriately, the screen time–related items fell in this cluster, including items “8. Limiting my child’s time using a phone or tablet” and “9. Limiting my child’s time watching TV.” Interestingly, the phone or tablet item had a higher importance rating than watching TV (4.00 compared to 3.67 out of 6), suggesting phones and tablets may be a particular area of focus for screen time strategies. Tips for screen time reduction were desirable among interview participants. Barriers included: apartment living or not having a yard, child injuries, busy schedules, younger children (eg, infants) in the household, child health issues, and caregiver being unconcerned about screen time.

Similarly, the only sleep-related item, “15. Getting my child to bed on time,” fell into this cluster. Most interview participants described a bedtime routine for their young children and stated that their child had a set bedtime. Participants who stuck to a routine described their child’s sleep and sleep compliancy as easy, with good quality and quantity. Alternatively, some caregivers described children who had difficulty falling asleep, resisted sleep, or had issues with waking regularly in the night. Several sleep improvement strategies included having a light, night light, or red light in the room; reading to children before bed; use of supplemental melatonin; soft music; and limits on screen time. Participants also described barriers, such as the use of screens, continuation of breastfeeding, and co-sleeping.

### Creating Healthy Eating Habits

The food and nutrition items grouped together as its own cluster (Creating Healthy Eating Habits), including a variety of eating and meal planning or preparation items. The most highly rated were as follows: “3. Having a variety of healthy food choices at home” (4.63 out of 5), followed by “12. Being a good role model for my child by eating healthy” (4.42 out of 5) and “19. Getting my child to eat vegetables” (4.38 out of 5). Mealtime and child feeding were major themes of the interviews. Participant responses were mixed if planning healthy meals was easy, difficult, or somewhere in-between. Many participants stated they actively try to prepare and provide healthy meals for themselves and their children. However, barriers to planning healthy meals included busy schedules or time, finances, food allergies or intolerances in the household, caregivers identifying as poor cooks, and having to tend to more than one young child. Convenience food and eating out were mentioned by parents as a strategy to address busy schedules.

Barriers to desirable child feeding included the child wanting to eat different foods than those prepared, frequent snacking, and wanting to eat close to bedtime. Picky eating was the most frequently raised barrier; only 5 caregivers stated their child was not a picky eater. Successful strategies to combat picky eating included eating meals with children or modeling healthy eating, cooking or preparing food with children, making food child-friendly, and keeping foods separated or compartmentalized on the plate or during serving. Unsuccessful strategies included food coloring or telling the child that a favorite character or animal eats the food. Many parents also stated encouraging vegetable eating was an important part of child feeding. Notably, item “21. Ways to address picky eating” had the second-lowest importance rating in the cluster (3.50 out of 5); this rating may indicate participant preferences toward broadly increasing positive strategies and healthy choices rather than a focus on specific strategies geared toward picky eating.

Some parents practiced strict restriction of what they considered unhealthy or junk foods, while others allowed children to have unimpeded access to snacks high in sugar, fat, and excess calories. A few caregivers expressed concern over their own weight and how this extended to their concern about child overweight or obesity. Although located in the Building Good Relationships cluster, participants highly rated item “23. Helping my child feel good about their body as they grow up” (4.88 out of 5), further emphasizing participant concerns about weight and body image as it relates to food choices.

The diversity of thought around meal planning and grocery shopping may be reflected by the lack of highly rated items. For example, items such as “16. Preparing meals at home,” “17. Cooking with my child,” and “20. Quick and easy recipes for my child” that correspond to planning and preparing meals fell at the bottom of the ratings for the Creating Healthy Eating Habits cluster (3.92, 3.58, and 3.50 out of 5, respectively). The relatively lower ratings of these items may be due to the wide range of opinions and strategies used by study participants. Participants who identified as meal planners described their motivations and strategies, including budgeting, shopping for staples, or sticking to the list. Some parents found it easier to buy only a few items at a time, while others found it easier to shop in bulk—using storage capacities of their pantries and freezers. Key facilitators included proximity to the grocery store, sharing tasks with another parent or caregiver, and allowing children to choose items at the store. Barriers included busy schedules, lack of time, finances, child misbehavior, and shopping with children present.

#### App Features

**Figure 2:**
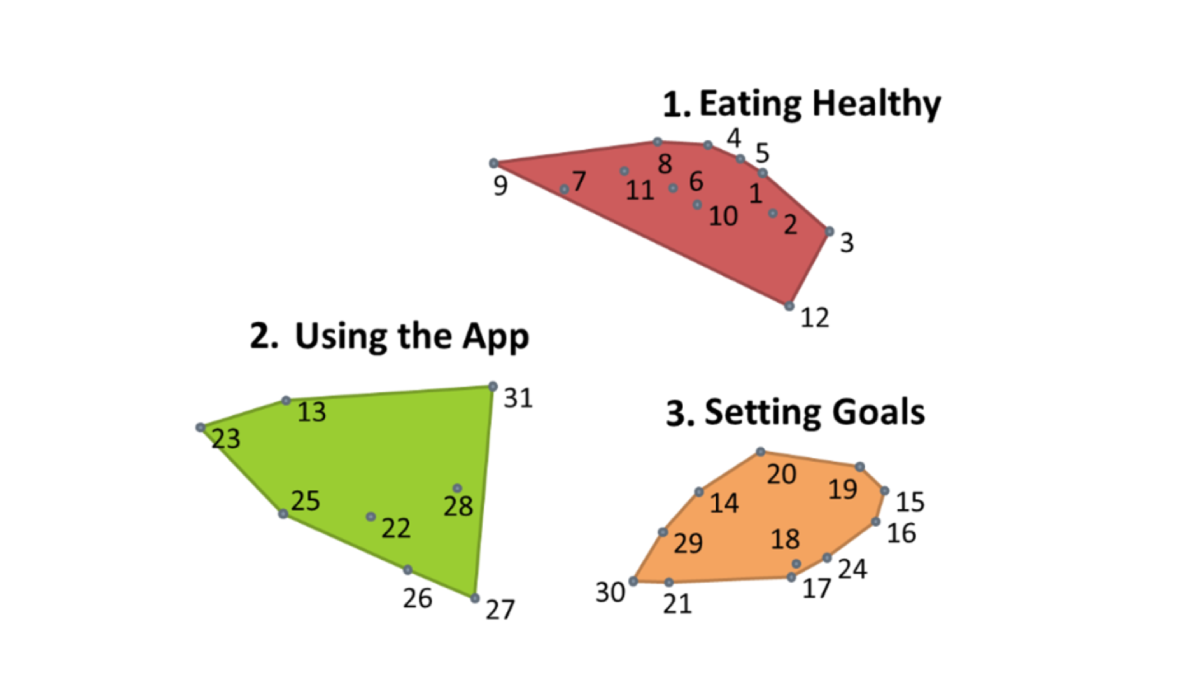
Application features combined point and cluster concept map.

Figure 2 shows the combined point and cluster map for the App Features area. The App Features map included the following clusters: (1) Eating Healthy, (2) Using the App, and (3) Setting Goals; likewise, these cluster names were developed from participant pile names in the sorting activity. In the interviews, participants were asked about their current use of apps, especially those that helped with parenting. The type of apps most frequently used by participants to help with parenting were social media (eg, Facebook, Instagram, Snapchat), which caregivers used to follow nutrition-related content, such as recipes, and for parenting advice or finding childcare. Many parents said they used apps to search for and view recipes, including through social media pages, Pinterest, YouTube, and WIC apps. Other participants preferred to use general search engines to find recipes.

### Eating Healthy

The concept mapping results support a high interest level in recipes. Items “1. Healthy recipes” (4.42 out of 5), “4. Easy recipes” (4.29 out of 5), and “5. Quick recipes” (4.29 out of 5) had the highest ratings in the Eating Healthy cluster, indicating participants are likely to use these features often or all the time. Recipe viewing was among the most popular desired app features in the interviews; however, some participants did mention they either do not use recipes or are not interested in looking up recipes in an app feature. Participants expressed interest in recipes for the entire family, which could include kid-friendly options, rather than child-specific recipes. Caregivers’ desire for planning features like a grocery list maker and meal planning tools was also desired; however, in the concept mapping results, items around meal planning and grocery lists were not as highly rated as those related to recipes. When discussing this as an app feature, participants explained how meal or grocery shopping features may be more likely to be used if they are kept simple.

### Setting Goals

The concept mapping findings also support the popularity of goal-tracking features. Item “20. Set and track goals about making food more child-friendly” (3.88 out of 5) had the highest rating for potential use among a variety of goal-tracking items in the Setting Goals cluster. Tracking features, especially goal tracking (eg, healthy eating, sticking to a routine, sleep, etc.) were also among the most desirable features among interview participants. Alternatively, some caregivers mentioned that, due to a lack of time or disinterest, they do not wish to log items or meticulously keep track of child-related goals on their phone. Some parents said this type of activity is already intuitive for them, so the feature would be unnecessary. Other participants said they already use a form of goal tracking that the app would allow them to simplify.

### Using the App

Items “13. App alerts to view tips about child health and nutrition” (3.50 out of 5) and “31. Links to views about child health and nutrition” (3.50 out of 5) had the highest ratings among the Using the App cluster, supporting participants’ desire for helpful tips and notifications. The next highest rated item was “23. Configure app preferences,” suggesting that participants would like to tailor the app settings to their preferences. In the interviews, helpful tips and notifications were welcomed by caregivers; however, many wanted to be clear about the desired frequency at which they wished to be notified on their phone. Developmental milestone tracking, general parenting tips, and the desire for everything to be in one place were desirable app features. Resoundingly, caregivers also voiced that simplicity and efficiency were important to them in an app.

#### Synthesis of Parental Priorities and App Features

Table 4 presents a list of possible content and features for a novel mobile app to improve health behaviors among preschool-aged children, based on the combined interview and concept mapping results. The first column depicts the topic areas of the overarching interview codes, the second column lists possible app content areas based on interview subcodes for parental priorities, and the third column lists possible app features based on interview subcodes along with the features rated as most desired in the concept mapping data, which are indicated in bold italics. By first understanding parental priorities, we ensured our resulting app content will be grounded in user-centered needs. In particular, parental feedback pointed to the need for a holistic app with a variety of health topics in one place, including a strong focus on food and nutrition, routines and time management, along with general parenting tips, each of which correspond to the clusters from the concept mapping process (Creating Healthy Eating Habits, Forming Boundaries, and Building Good Relationships). Informed by the concept mapping rating data, we gained information on ways to focus the development of our novel mobile app through the inclusion of quick, easy, and child-friendly recipes; grocery list or meal planning tools; goal tracking; helpful parenting tips; and the ability to control settings, such as notifications and recipe filters, to customize the experience for individual users. As a whole, exploring parental priorities and mobile app features in combination ensured our app development will be informed by both the desired content and functions that target users (parents with preschool children) would like to have.

**Table 4:** Summary of possible app content and features based on interview and concept mapping results (most desired features in italics).

Interview topic	Parental priorities: possible app content	Parental interests: possible app features
Food and nutrition	• Mealtime or eating behavior (eg, picky eating) • Making/planning healthy meals	• *Quick, easy recipes*: Kid-friendly; cooking with kids Sort or filter ingredients; allergies • Grocery list or meal planning • Picky eating and child nutrition tips
Other health-related	• Limiting screen time • Dental health • Activities for children	• *Desire for multiple health topics in one place (holistic)* • Active play and activities for children tips • Features for parent and child
Routine and time management	• Time management/planning • Managing work responsibilities and parenting	• *Goal tracking* • *Ability to control settings* • Sleep (bedtime/nap) tracking • Ability to share success
Behavioral issues	• Discipline and rule setting • Sticking to boundaries • Reacting mindfully to behavior and patience • Influencing your child	• *General parenting tips* • Interaction with other parents or users • Account for other family members or parent
Developmental milestones	• Potty training • Improving speech • Socialization and interacting with others • Improving sensory development	• Developmental milestone tracking • Communication with health care provider

## Discussion

### Principal Results and Comparison With Prior Work

Concept mapping has been used on projects focused on childhood obesity prevention programs for adolescents [36] or for the development of culturally appropriate interventions [37], as well as for areas such as sugary drink consumption among children [38], food parenting practices [39], and overall childhood thriving [40]. To our knowledge, however, our study is the first application of the method to guide the development of a health behaviors app for parents of preschool-aged children. In this study, we used a novel, convergent mixed methods approach to identify parental child health priorities and mobile app features that parents or caregivers would prefer to use. Through the use of qualitative interviews, we were able to describe the breadth of parenting challenges and experiences the participants faced. With the addition of concept mapping activities, we were able to identify converging themes and assess priorities among the provided information.

Overall, the participants in this study indicated psychosocial health and general wellbeing, rather than specific behaviors, as their primary focus when expressing their interest in improving health among their preschool-aged children for mobile app content. Using previous research, 12 behavioral factors were identified related to childhood overweight and obesity that parents identified when thinking about the health of their child [10,11]. Parent and caregiver responses in this study suggest the importance of establishing a routine and setting boundaries, particularly in areas such as screen time and sleep behaviors. Although healthy eating arose as the most desired topic for resources for young children, participants described a wide variety of preferences around meal planning, grocery shopping, and ways to improve food-related behaviors. Notably, our participants described the importance of all of the related areas identified in the previous literature as important app content to building healthy behaviors for preschool-aged children, including nutrition, physical activity, media use, and sleep.

For specific app features, parents indicated their top choices as (1) quick, easy, and child-friendly recipes; (2) goal-tracking features; and (3) the use of tips and notifications. Goal-setting has been a common behavior change feature used in mobile health applications for children in previous studies with techniques used such as rewards for making progress [18,41,42]. However, there has been a significant gap in that many of the mobile health applications do not feature the involvement of health professionals [18], and few apps exist with the intention of parents using them to modify their children’s health behaviors [16,17,43]. In the recent review on goal-setting applications for parents and children, 9 applications were identified that allowed goal-tracking in the setting of health-related behaviors of children, with 6 focused on nutrition or mealtime, 5 focused on physical activity or screen time, 7 focused on sleep, and 6 focused on personal hygiene. None of the apps allowed a parent to specifically recommend goals for each child [17]. Furthermore, matching the divergent views about healthy eating strategies, our participants expressed diversity of thought on their potential use of meal planning or grocery shopping features, though many indicated they would use such features in an app. Additionally, participants expressed interest in tailoring the tips or notifications to a variety of use preferences. As with the health behaviors, our participants described a desire to have everything in one place, so they can find what they need quickly and easily depending on specific needs for their child.

Nutrition and facilitating family mealtimes and daily routines were clearly identified as priorities for this sample of parents and caregivers. The implications of the diverse needs, preferences, and priorities for child health described here may indicate that utility and engagement with mobile tools rest in the balance of simplicity with a choice of multiple features and content foci. Shopping, recipes, and cooking were central to the concerns expressed by these individuals. App features focused on recipes may be designed to incorporate multiple needs and priorities. For example, in order to support the “ease” of healthy meal preparation, provide a recipe feature that allows filtering by time to prepare, provide recipes that address different dietary requirements based on allergies or cultural preferences, or make a shopping list easy to create based on items needed for recipes. Given the preference for a one-stop mobile app, we anticipate that parents will be more engaged with apps that incorporate the whole child and provide behavioral, social, and psychological wellness resources as well.

### Limitations

The study sample is representative of relatively lower income families who use public assistance programs; however, the sample included primarily non-Hispanic white and married participants whose perspectives may not represent those of other ethnicities or demographic groups. Future studies in this area should recruit and stratify participants of diverse backgrounds, including single parents and non-traditional family structures, to uncover differences in needs or desires. In addition, individuals choosing to participate in this type of study may be more engaged with parenting practices and improving their child’s health than the general population, resulting in selection bias. The use of technology, such as Zoom, can also be a concern among this population; as such, we provided one-on-one assistance to ensure participants felt comfortable using the platform. Additionally, the study had a relatively small sample size, although appropriate for a qualitative approach. Likewise, the purpose of concept mapping is to identify consensus but is dependent upon the composition of the participant sample. Within the concept mapping data, high importance ratings with low variability are often seen, as items are included in the brainstorming list based on their potential importance; we experienced this issue in our data. However, by using multiple methods to triangulate our findings, we feel confident in the ability of our results to accurately capture the perceptions of parents of young children.

### Conclusions

For our team, this formative research provided the groundwork for the development of a novel mobile app, including both content and features, for parents and caregivers of preschool-aged children focused on guided goal setting across the domains of diet, physical activity, media use, and sleep. A convergent mixed methods approach provided high-quality data on diverse parental perceptions and challenges and needs for families. Specific app features identified to meet family needs should be designed closely with a diverse set of families and tested using rigorous designs to identify the mechanisms of action that mobile apps may use for efficacious healthy parenting outcomes. This study makes important contributions to the mHealth field for understanding what parents or caregivers of young children want from mobile apps to support building healthy behaviors and routines. The findings can inform future research on the development and evaluation of existing or new mobile apps.

## Appendix

Multimedia Appendix 1 Semistructured discussion guide for interview questions.

## Abbreviations

mHealth: mobile health
REDCap: Research Electronic Data Capture
SNAP: Supplemental Nutrition Assistance Program
WIC: Special Supplemental Nutrition Program for Women, Infants, and Children
