# Supplementary figures and images for: Parental Perceptions of Priorities and Features for a Mobile App to Promote Healthy Lifestyle Behaviors in Preschool Children: Mixed Methods Evaluation
Source: JMIR Pediatr Parent. 2025 Feb 19;8:e65451. doi: 10.2196/65451 (PMC11888088; doi:10.2196/65451)

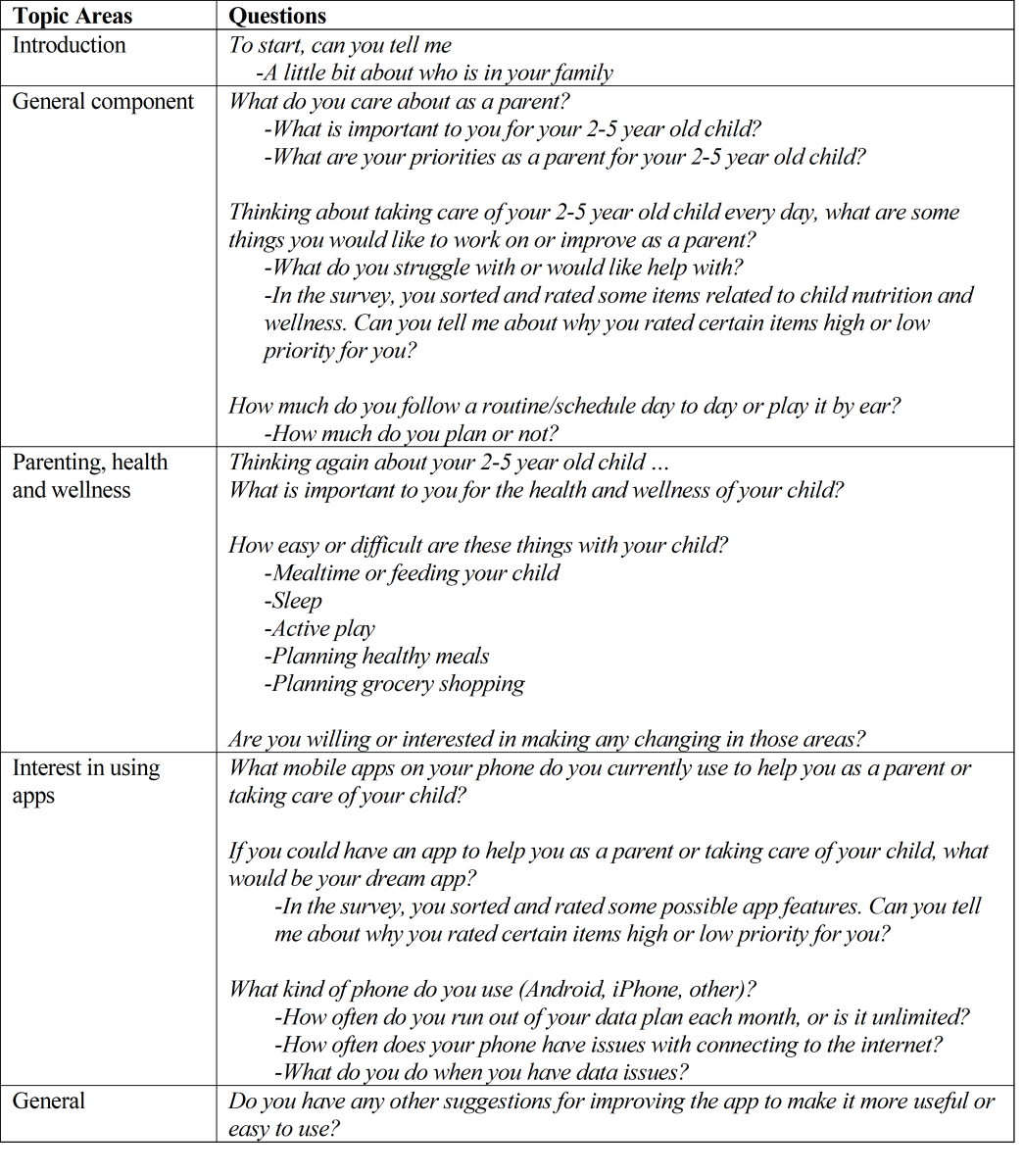

Supplement: Multimedia Appendix 1 [file pediatrics_v8i1e65451_app1.png]
